# Spatial imaging of Zn and other elements in Huanglongbing-affected grapefruit by synchrotron-based micro X-ray fluorescence investigation

**DOI:** 10.1093/jxb/ert450

**Published:** 2014-01-13

**Authors:** Shengke Tian, Lingli Lu, John M. Labavitch, Samuel M. Webb, Xiaoe Yang, Patrick H. Brown, Zhenli He

**Affiliations:** ^1^University of Florida, Institute of Food and Agricultural Sciences, Indian River Research and Education Center, Fort Pierce, FL 34945, USA; ^2^MOE Key Laboratory of Environment Remediation and Ecological Health, College of Environmental and Resource Science, Zhejiang University, Hangzhou 310058, China; ^3^Department of Plant Sciences, University of California, Davis, CA 95616, USA; ^4^Stanford Synchrotron Radiation Lightsource, SLAC National Accelerator Laboratory, Menlo Park, CA 94025, USA

**Keywords:** Candidatus Liberibacter asiaticus, distribution, Huanglongbing, grapefruit, phloem, micro-XRF, zinc.

## Abstract

Huanglongbing (HLB) is a highly destructive, fast-spreading disease of citrus, causing substantial economic losses to the citrus industry worldwide. Nutrient levels and their cellular distribution patterns in stems and leaves of grapefruit were analysed after graft-inoculation with lemon scions containing ‘*Candidatus* Liberibacter asiaticus’ (Las), the heat-tolerant Asian type of the HLB bacterium. After 12 months, affected plants showed typical HLB symptoms and significantly reduced Zn concentrations in leaves. Micro-XRF imaging of Zn and other nutrients showed that preferential localization of Zn to phloem tissues was observed in the stems and leaves collected from healthy grapefruit plants, but was absent from HLB-affected samples. Quantitative analysis by using standard references revealed that Zn concentration in the phloem of veins in healthy leaves was more than 10 times higher than that in HLB-affected leaves. No significant variation was observed in the distribution patterns of other elements such as Ca in stems and leaves of grapefruit plants with or without graft-inoculation of infected lemon scions. These results suggest that reduced phloem transport of Zn is an important factor contributing to HLB-induced Zn deficiency in grapefruit. Our report provides the first *in situ*, cellular level visualization of elemental variations within the tissues of HLB-affected citrus.

## Introduction

Huanglongbing (HLB, or citrus greening) is a highly destructive, fast-spreading disease of citrus caused by phloem-limited, non-cultured, Gram-negative liberibacters (α-Proteobacteria; Bove, 2006). Three forms of HLB bacteria have been identified: a heat-tolerant form, ‘*Candidatus* Liberibacter asiaticus’; a heat-sensitive African form, ‘*Ca.* L. africanus’; and a heat-sensitive form, ‘*Ca.* L. americanus’, that is found in Brazil (Coletta *et al.*, 2004; Bove, 2006). So far, *Candidatus* Liberibacter asiaticus (Las) is the form that has contributed most to the spread of HLB. Citrus HLB has been reported in many countries; for example, China, Brazil, United States (Florida), India, Iran, Cuba, the Dominican Republic, and Ethiopia (Faghihi *et al.*, 2009; Martinez *et al.*, 2009; Matos *et al.*, 2009; Das and Kumar, 2010; Manjunath *et al.*, 2010; Saponari *et al.*, 2010). It is the most destructive of all citrus pathosystems worldwide; there is nowhere in the world where HLB is found where it is under adequate control (Gottwald, 2010). The rapid spread of HLB in Brazil and Florida, where citrus juice production accounts for over one-third of the world’s output (Manjunath *et al.*, 2008), has brought renewed interest in the disease due to its potentially devastating impact on the citrus industry. Citrus HLB was detected in Florida in 2005; it spread quickly and now threatens to devastate the entire 640 000 citrus-planted acres in the state (Etxeberria *et al.*, 2009). Las-caused HLB also constitutes a very serious problem for the Chinese citrus industry; for example, 67.1% of the citrus fruit harvested from all 12 cities in Guangdong Province were positive for Las, with 15 of the 16 cultivars planted affected (Deng *et al.*, 2012).

Typical HLB symptoms are the production of abnormal-looking fruit and an often blotchy chlorosis of the leaves, these symptoms are followed by tree decline and death in the advanced stages (Albrecht and Bowman, 2011). The most severe problem for HLB-infected citrus plants is the inhibition of the phloem transport of photoassimilates and the subsequent altered carbon partitioning. Consequently, extraordinary levels of starch accumulate in leaves; this results in imbalances in carbohydrate partitioning that affect the overall health of diseased trees (Etxeberria *et al.*, 2009). The reported off-flavour associated with symptomatic juices has been suggested to stem from lower concentrations of sugars (Dagulo *et al.*, 2010). Excessive starch build-up is also believed to cause disruption of the chloroplast thylakoid system, thus leading to the mottled, chlorotic leaf symptoms (Etxeberria *et al.*, 2009). Nevertheless, a recent report (Liao and Burns, 2012) has suggested that development of HLB symptoms might also be associated with the host’s disease response rather than being solely a direct consequence of carbohydrate starvation.

Minerals are important elements for biochemical and physiological processes in plants (Marschner, 1995) and the leaf symptoms of HLB-infected plants, including vein yellowing and mottled leaves, generally resemble symptoms of mineral nutrient deficiencies (Liao and Burns, 2012). For instance, symptom-based identification of HLB-affected trees is difficult due to foliar symptom similarities with Zn deficiency (Albrecht and Bowman, 2008; Cevallos-Cevallos *et al.*, 2011). It is thus relevant to determine if element distribution differences exist between the HLB-affected and healthy plants. Koen and Langenegger (1970) found that concentrations of K were higher, while Ca and Mg were lower in an unnamed citrus species infected with the *Ca.* L. africanus-caused form of HLB. Aubert (1979) showed that infected plants in Réunion contained lower concentrations of Ca, Mn, and Zn. Several other reports also indicated that the application of mineral fertilizers alleviated the symptoms of HLB-affected trees. For instance, an application of Zn or Cu ions in combination with Ca was able to delay HLB disease incidence and severity, resulting in a significant increase (*P* ≤0.05) in fruit production (Ahmad *et al.*, 2011). Furthermore, foliar fertilization, including several mineral elements (Zn, Fe, Ca, K, and Mn) reduced HLB symptom expression of infected trees (Pustika *et al.*, 2008).

Despite the similarity of HLB and mineral deficiency symptoms (Zn, in particular), there have been few studies on elemental variation in HLB-affected citrus plants; thus leaving open the question of possible mineral deficiency contributions to the appearance of HLB disease symptoms or vice versa. In the present study, grapefruit (*Citrus paradisi*) plants were graft-inoculated with Las-infected lemon (*Citrus limon*) scions. After 12 months, mineral nutrient levels in healthy (control) and HLB-affected grapefruit were analysed by ICP-MS and compared. To understand potential HLB-influenced elemental distribution variations at the cellular level, distribution patterns were further analysed in both healthy and HLB-affected plants by synchrotron-based X-ray fluorescence (µ-XRF). This technique has been widely used in the research of elemental distribution in plant tissues and has proved to be a promising tool to study the *in vivo* localization of metals in plants due to its high resolution and sensitivity (Lombi and Susini, 2009; Ahmad *et al.*, 2011; Regvar *et al.*, 2011; Koren *et al.*, 2013). XRF analyses can be performed without much pretreatment of the plant samples. This technique has previously been applied to investigate differential metal distribution patterns in a Zn/Cd co-hyperaccumulator *Sedum alfredii* ecotype (Tian *et al.*, 2010; Fan *et al.*, 2011; Masaoka *et al.*, 2011). Application of the sensitive µ-XRF technique in this study should shed light on possible interactions between mineral distribution status and HLB disease in citrus plants.

## Materials and methods

### Plant culture

Three-year-old healthy grapefruit seedlings were graft-inoculated with Las-infected lemon scions and were subsequently maintained in the greenhouse. After 12 months, the typical HLB symptoms (vein corking and mottled leaves) were evident on the leaves of the inoculated grapefruit seedlings. The citrus seedlings with typical HLB symptoms were tested for the presence of ‘*Ca.* L. asiaticus’ bacteria and the presence and distribution of selected mineral nutrients.

### Genomic DNA extraction and qPCR analysis

Genomic DNA extraction and qPCR analysis of plant samples were performed according to Zhang *et al.* (2011). Plant samples were rinsed three times with sterile water. DNA was extracted from 0.1g of plant samples (fresh weight) using Qiagen’s DNeasy Plant Mini Kit (Qiagen, Valencia, CA). qPCR was performed with primers and probes (HLBas, HLBr, and HLBp) for ‘*Ca.* L. asiaticus’ using the ABI PRISM 7500 sequence detection system (Applied Biosystems, Foster City, CA) in a 20 µl reaction volume consisting of the following reagents: 300nM (each) target primers (HLBas and HLBr), 150nM target probe (HLBp), and 1× TaqMan qPCR Mix (Applied Biosystems). All reactions were performed in triplicate and each run contained negative (DNA from healthy plants) and positive (DNA from HLB-affected plants) controls. Data were analysed using the ABI 7500 Fast Real-Time PCR System with SDS software. The cycle threshold (Ct) values were converted to estimated bacterial titres using the grand universal regression equation *Y*=13.82–0.2866 *X*, where *Y* values are the estimated log concentrations of templates and *X* values are the qPCR Ct values. Plants were considered to be PCR negative for Las when the Ct values were >36.0, which is equivalent to an estimated bacterial titre of <1 60 cells g^–1^ of plant tissue.

### Measurement of nutrient elements in leaves

The leaves of healthy and HLB-affected grapefruit were oven-dried at 65 °C for 72h. The dried plant materials were then ground using a stainless steel mill and passed through a 0.25mm sieve for the analysis of nutrient elements. Ground, dry plant samples (0.1g) of each treatment were digested with 5.0ml HNO_3_–HClO_4_ (4:1, v/v), and the digest was transferred to a 50ml volumetric flask, made up to volume with water and filtered. Concentrations of mineral elements (i.e. Zn, Fe, Cu, Mn, Ca, K, Mg, and P) in the filtrates were analysed using inductively coupled plasma mass spectroscopy (ICP-MS) (Agilent 7500a, USA). Phosphorus content was analysed by the molybdenum blue method after digestion with H_2_SO_4_–H_2_O_2_ at 300 °C.

### Elemental mapping of stems and leaves by µ*-*XRF

Fresh stems and leaves were cut from plants and rinsed with deionized water. Different stem and leaf samples at similar developmental stages were selected from HLB-affected and control plants for comparisons. Sections (40 µm thick) of samples were cut with a cryotome (Leica, CM1950) at a temperature of –20 °C, as by Tian *et al.* (2010). Micro-XRF imaging was performed on at the Stanford Synchrotron Radiation Laboratory (SSRL) using Beam Lines 10–2 and 2–3. Experiments on Beam Line 10–2 were recorded at 13 500eV, using a 20 µm (H)×20 µm (V) beam spot size, a 20 µm×20 µm pixel size, and 100ms dwell time per pixel. The incident X-ray beam of 2 µm in Beam Line 2–3 was focused using a pair of Kirkpatrick–Baez mirrors, and the incident beam was monochromatized using a Si(111) double-crystal monochromator. Micro-XRF maps were obtained by rastering the beam at 5 µm steps, with a count time of 200ms per step, for the following major and minor/trace elements: P, S, Cl, K, Ca, Mn, Fe, Ni, Cu, and Zn. Fluorescence signal intensities for the above elements were translated to concentrations (µg cm^–2^) in the cross-sections of each plant sample for semi-quantitative analysis. Known XRF calibration standards mounted on 6 µm thick mylar film (Micromatter, Vancouver, Canada), were imaged under the same conditions as the samples. Element concentrations were calculated in SMAK software (Webb, 2006) by using measurements of the standards to obtain counts per second per µg cm^–2^, and then dividing by the pixel size to yield element concentrations. The fluorescence data were presented as tricolour maps that allow the spatial distributions of three elements to be shown. Pixel brightness was displayed in RGB, with the brightest spots corresponding to the highest element fluorescence.

### Statistical analysis of data

All data were statistically analysed using the SPSS package (Version 11.0). Analysis of variance (ANOVA) was performed on the data sets and the mean and SE of each treatment as well as LSD (*P* <0.05 and *P* <0.01) for each set of corresponding data were calculated.

## Results

### Plant growth and HLB diagnosis

**Fig. 1.**
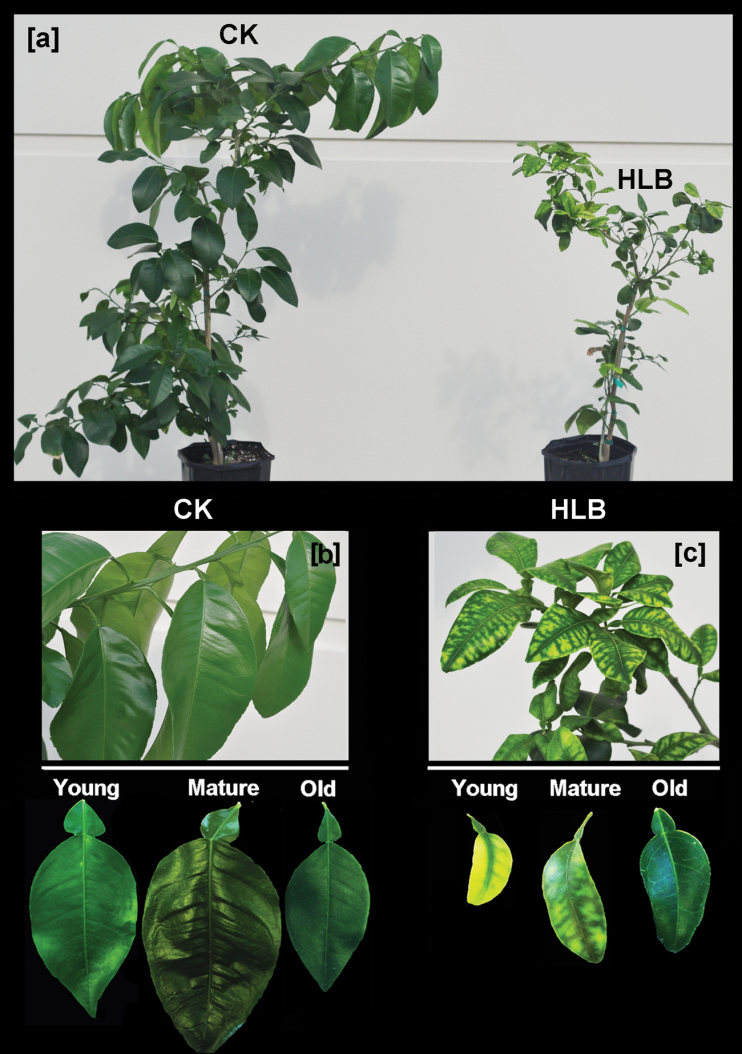
(a) Huanglongbing (HLB)-affected (right) and healthy control (left) grapefruit plants. Photograph was taken 12 months after the graft inoculation with Las-infected lemon scions. Typical HLB symptoms of curled and mottled leaf blades were apparent (c) compared with healthy trees (b).

**Fig. 2.**
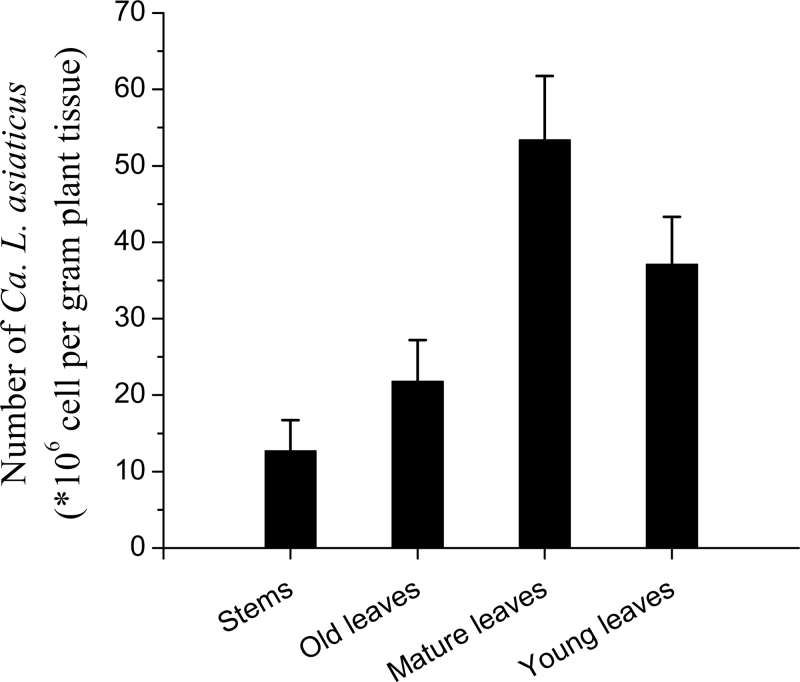
‘*Candidatus* Liberibacter asiaticus’ bacterial titres (×10^6^ cells g^–1^ of plant tissue) in the stems and young, mature, and old leaves collected from grapefruit plants 12 months after graft inoculation.

Twelve months after graft-inoculation of grapefruit plants with Las-infected lemon scions, the HLB-affected grapefruit plants showed stunted growth when compared with the healthy control trees (Fig. 1a). Typical HLB symptoms (curled, mottled chlorotic leaves that were smaller than leaves on healthy plants; Fig. 1b, c) were observed. PCR was used to identify ‘*Ca.* L. asiaticus’ in all plants that had been grafted with the Las-infected lemon scions (Fig. 2), thus confirming the HLB diagnosis.

### Concentrations of Zn and other minerals

Concentrations of nutrient elements, including Zn, Fe, Mn, Cu, Ca, K, Mg, and P, in the tissues of HLB-affected grapefruit plants were determined by ICP-MS and compared with the controls (Table 1). In both the healthy and HLB-affected plants, Zn concentrations in old and mature leaves were higher than those in the young leaves and Zn concentrations in all of the grapefruit leaves, regardless of their maturity, were significantly reduced in HLB-affected leaves compared with the healthy leaf samples. The concentrations of other elements in the leaves of grapefruit plants varied in response to Las infection. Infection decreased Mn and Cu accumulation in the young, but not mature and old leaf samples, but there was no significant infection impact on Fe accumulation for leaves of any maturity. No significant HLB-induced variation was found for macronutrients (i.e. K, Ca, Mg, and P) in the young, mature, and old leaves of grapefruit, with the possible exception of a slight decrease of P in the old, Las-infected leaves.

**Table 1.** Concentrations of Zn, Fe, Mn, Cu (mg kg^–1^ DW) and Ca, K, Mg, P (mg g^–1^ DW) in young, mature, and old leaves collected from healthy (CK) and HLB-affected grapefruit plantsOne or two asterisks represent the least significant differences (*P* <0.05 and *P* <0.01, respectively) between leaf samples on a healthy tree and a sample with the corresponding age from an HLB-affected tree. Data points represent means from three individual plants.

Plant samples		Zn	Fe	Mn	Cu	Ca	K	Mg	P
		mg kg^–1^ DW				mg g^–1^ DW			
CK	Young	34.6±4.5*	73.7±9.6	73.0±4.1*	16.7±2.0**	12.0±1.3	1.48±0.08	2.32±0.35	12.3±1.1
	Mature	38.7±2.6*	67.9±8.7	66.8±8.6	11.9±1.8	13.2±1.5	1.62±0.08	2.71±0.27	14.6±0.9
	Old	42.4±5.1*	78.8±9.1	87.9±8.1	17.9±4.3	26.3±2.7	2.23±0.13	3.86±0.84	11.5±0.9*
HLB	Young	24.1±2.9	71.4±8.6	51.0±6.2	8.9±1.5	14.5±1.2	1.31±0.21	2.66±0.13	14.9±1.3
	Mature	26.7±3.1	70.6±6.4	52.6±5.8	9.5±3.2	14.3±1.4	1.27±0.10	2.37±0.13	13.4±1.1
	Old	32.8±4.3	86.2±8.7	80.7±7.5	15.1±2.5	28.7±3.7	1.89±0.19	5.08±0.62	8.5±0.9

### Spatial imaging of Zn and other elements in leaves

Distribution patterns of Zn and other elements in the cross-sections of stems and leaves collected from healthy and HLB-affected grapefruit plants were analysed by µ-XRF mapping at SSRL. The integrated intensities of Zn, K, Ca, Mn, Fe, Cu, P, and S were calculated from X-ray fluorescence spectra and normalized by I0 and the dwell time (data not shown). Elemental mapping for the measurement area was obtained from the normalized intensity for each element.

**Fig. 3.**
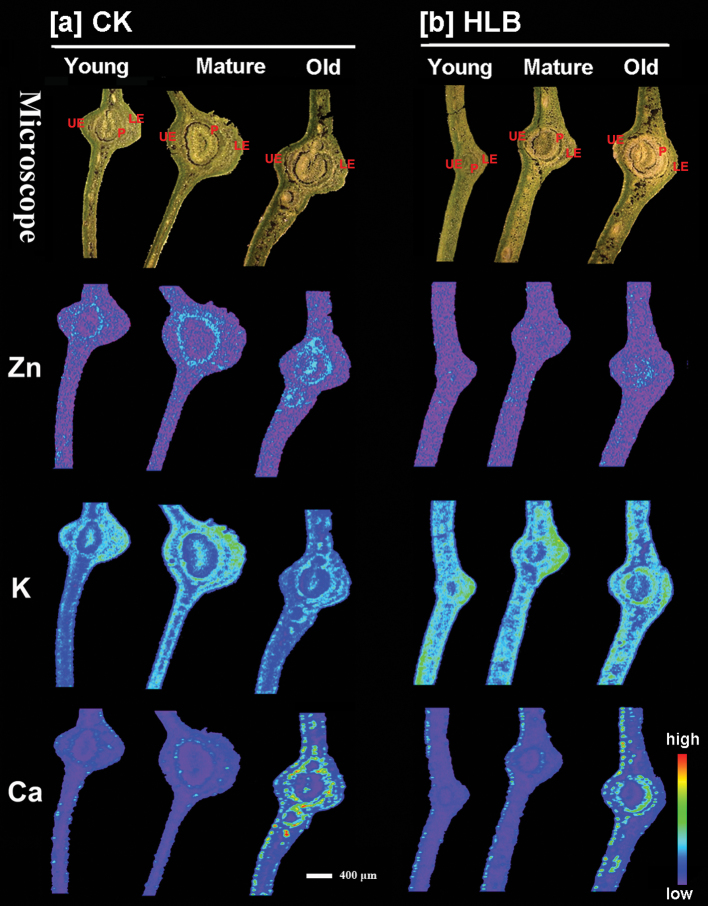
Micro-XRF mapping of elements (Zn, K, and Ca) in the cross-sections of young, mature, and old leaves collected from healthy (a) and HLB-affected (b) grapefruit plants. The number of fluorescence yield counts was normalized by I0 and the dwell time. The red colour, depicting elemental concentrations in each map, was scaled to the maximum value for each map. Scale bar: 400 µm. UE, upper epidermis; LE, lower epidermis; P, phloem.

Distribution patterns of Zn, K, and Ca in the cross-sections of young, mature, and old leaves are presented in Fig. 3. Elemental maps for the other elements are shown in Supplementary Fig. S1 available at *JXB* online. The normalized X-ray fluorescence intensities are scaled between red (maximum) and blue (minimum) for individual elements. As shown in Fig. 3, a significant difference was observed for Zn distribution patterns of leaf cross-sections between the healthy and HLB-affected plants. In the cross-sections of all the leaves (young, mature, and old) collected from healthy grapefruit plants (CK, Fig. 3a), Zn was preferentially distributed in the vascular system (specifically, phloem tissues), but this was not the case for Zn in HLB-affected leaves (Fig. 3b). Potassium and Ca distribution patterns in the leaf cross-sections of HLB-infected grapefruit were not significantly different from those of healthy plants.

**Fig. 4.**
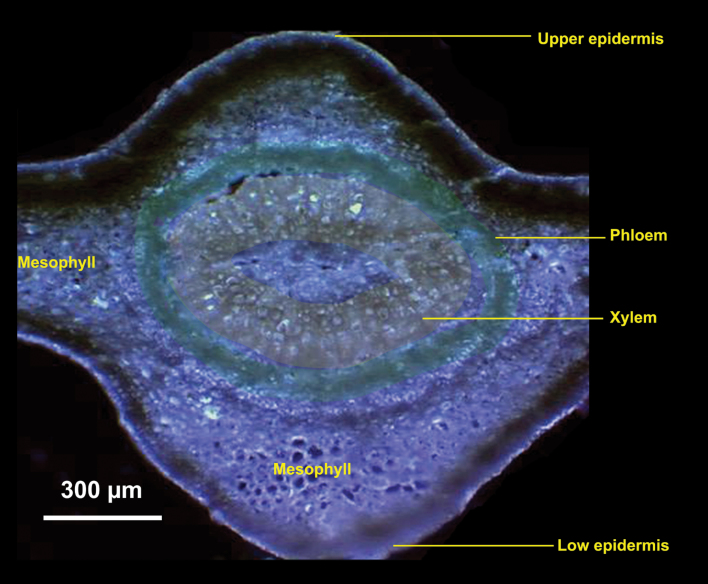
Microscope image of a cross-section of a mature leaf collected from a healthy grapefruit plant. Phloem tissues were marked with a green colour and the xylem was marked with a yellow colour. Scale bar: 300 µm.

**Fig. 5.**
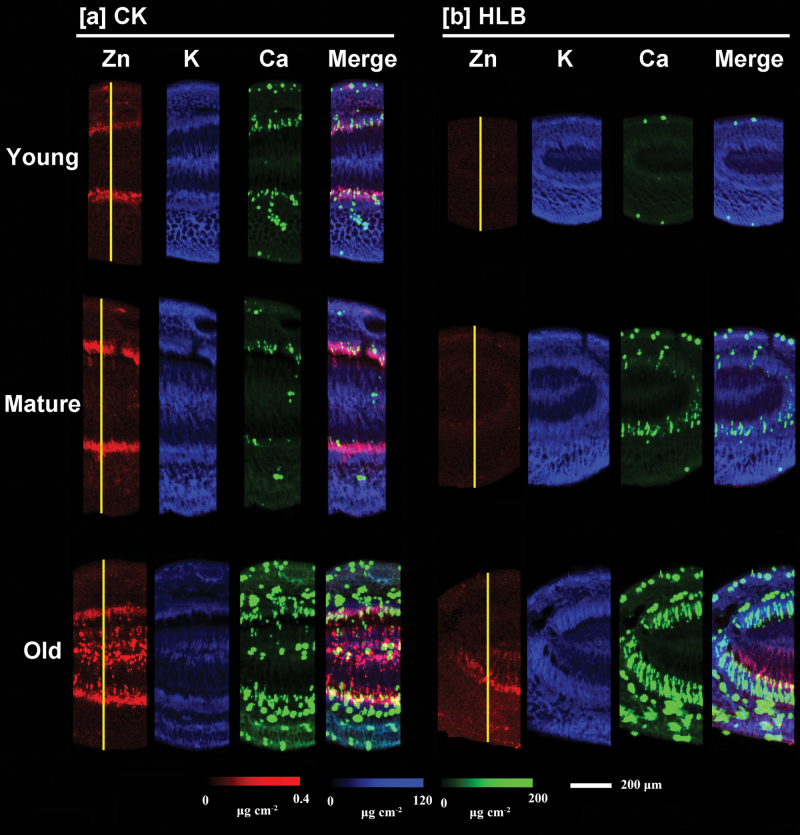
Micro-XRF mapping of Zn (red), K (blue), and Ca (green) in the cross-sections of young, mature, and old leaf blades collected from healthy (a) and HLB-affected (b) grapefruit plants. Fluorescence intensities of elements were normalized and subsequently translated to concentrations by using measurements of the standards. Pixel brightness is displayed in RGB, with the brightest spots corresponding to the highest concentrations (µg cm^–2^) for the element depicted. Scale bar: 200 µm.

To investigate in more detail the localization of elements in the leaf vascular systems (Fig. 4), µ-XRF was performed that focused on areas of leaf veins with a smaller step size of 2 µm (Fig. 5). On the basis of the intensities of elemental signals, the concentrations of the elements (µg cm^–2^) were calculated by using standard materials. Pixel brightness is displayed in RGB, with the brightest spots corresponding to the highest element fluorescence. These higher resolution elemental maps of the leaf vein area clearly showed that the intensity of Zn (red) was much higher in the cross-sections of healthy (CK; Fig. 5a), compared with infected leaves (Fig. 5b). The more intense Zn signals were associated with the phloem tissues of healthy leaves at all developmental stages, with an exception being the high Zn intensity observed in the centre parenchyma cells in the veins of healthy old leaves. However, in the leaf veins collected from HLB-affected grapefruit, Zn intensity was very low in young and mature leaves and no obvious preferential Zn distribution was noted in the phloem tissues.

**Fig. 6.**
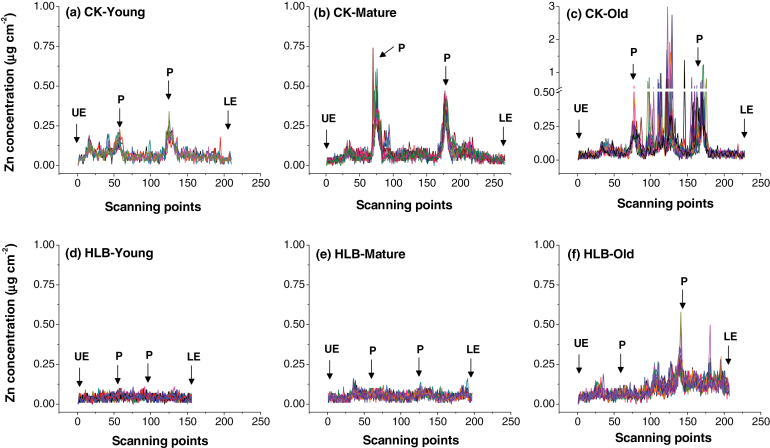
Zinc concentrations (µg cm^–2^) of the scanning sites selected to span the sections from the upper epidermis (UE) to the lower epidermis (LE) across the veins of young, mature, and old leaves collected from healthy (a, b, c) and HLB-affected (d, e, f) grapefruit plants. Note that the range of *y*-axis scale values in (c) is broken and, at its maximum, is three times that of the other scans. The selected scanning sites are marked by yellow lines in Fig. 4, with 10–14 different scanning lines selected for each plant sample. P, phloem.

Sections of young, mature, and old leaves collected from the healthy (CK) and HLB-affected grapefruit were scanned for Zn concentrations (µg cm^–2^) from the upper epidermis to the lower epidermis to determine the patterns of Zn cellular distribution as affected by HLB (Fig. 6). The scans clearly showed that Zn concentration was quite high (0.50–0.75 µg cm^–2^) in the phloem tissues of mature healthy leaves, a level several hundred-fold higher than that in the other tissues/cells of healthy leaves (Fig. 6b). By contrast, Zn intensity in mature, HLB-affected leaves was uniformly distributed throughout the tissue (Fig. 6e) and concentrations were less than 0.1 µg cm^–2^. The highest Zn intensity in healthy old leaf veins was observed in the central cluster of parenchyma cells, with up to 3.0 µg cm^–2^, with a second peak in the phloem tissues (Fig. 6c). By contrast, the intensity of Zn in the HLB-affected old leaves was significantly lower, although the preferential Zn localization was still in the centre and vascular tissues (Fig. 6f).

### Spatial imaging of Zn and other elements in stems

**Fig. 7.**
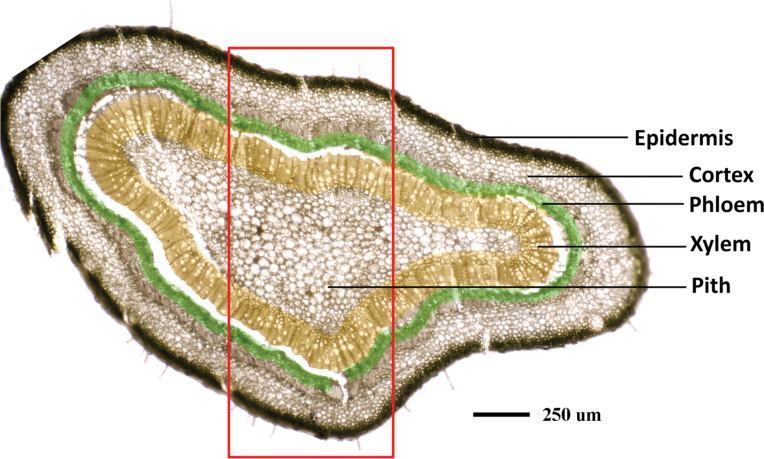
Microscope image of a stem cross-section collected from healthy grapefruit plants. Phloem tissues were marked with a green colour and the xylem was marked with a yellow colour. Scale bar: 250 µm.

**Fig. 8.**
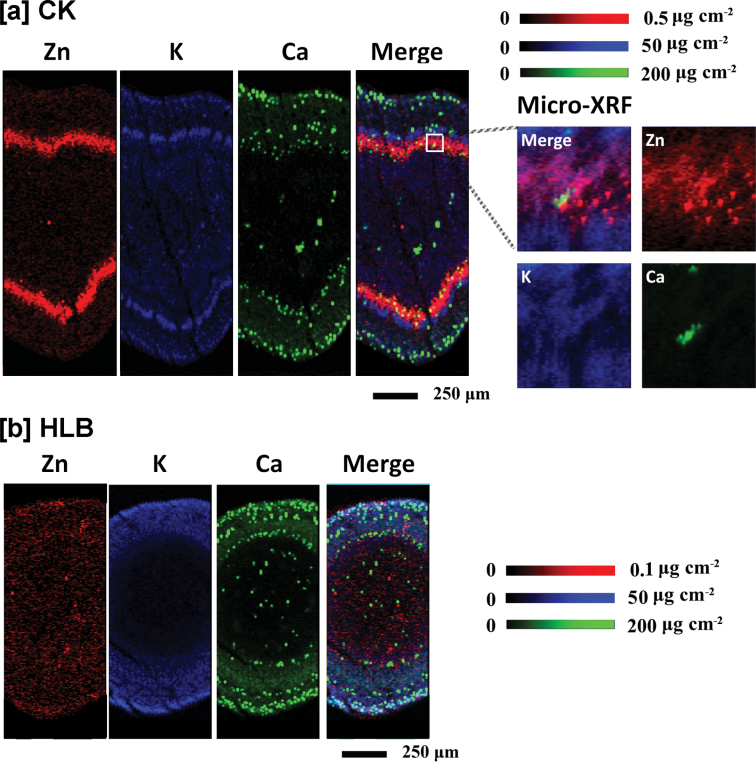
Micro-XRF mapping of Zn (red), K (blue), and Ca (green) in the cross-sections of stems collected from healthy (a) and HLB-affected (b) grapefruit plants. Fluorescence intensities of elements were normalized and subsequently translated to concentrations by using measurements of the standards. Pixel brightness is displayed in RGB with the brightest spots corresponding to the highest concentrations (µg cm^–2^) for the element depicted. Scale bar: 200 µm.

The stem consists of five major tissues from the outer layers toward the centre: the epidermis, cortex, phloem, xylem, and pith, as shown in Fig. 7. The selected areas of stem cross-sections (marked with the red box; Fig. 7) were subjected to µ-XRF imaging. The data for Zn concentration and distribution patterns are consistent with the results obtained with the scanned leaf sections (Figs 5, 6), with high Zn intensity almost exclusively localized in the phloem tissues of healthy stems (CK; Fig. 8a), as indicated by the very bright red colour, whereas Zn intensity was low and uniformly distributed throughout the HLB-affected stem (Fig. 8b). Preferential distribution of K in the phloem tissues was observed in the healthy stems (Fig. 8a) but not in the HLB-affected samples (Fig. 8b). The HLB-induced reduction of K in the phloem was not as significant as that observed for Zn. Distribution patterns of other elements, Ca, for example (Fig. 8a, b; others that were scanned for but which are not shown were Mn, Fe, Cu, P, and S etc.) were different from that of Zn, and no significant differences in these elements were observed between the healthy and HLB-affected stem tissues.

**Fig. 9.**
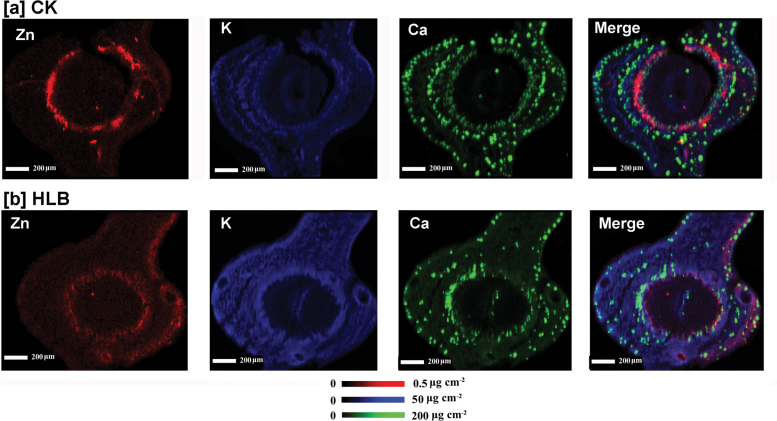
Micro-XRF mapping of Zn (red), K (blue), and Ca (green) in the cross-sections of stems collected from healthy (a) and HLB-affected (b) lemon branches. Fluorescence intensities of elements were normalized and subsequently translated to concentrations by using measurements of the standards. Pixel brightness is displayed in RGB with the brightest spots corresponding to the highest concentrations (µg cm^–2^) for the element depicted. Scale bar: 200 µm.

Spatial imaging of Zn and other elements was also performed on stem cross-sections collected from Las-infected lemon scions. The results showed that Zn was preferentially distributed to phloem tissues in the healthy stem sample (Fig. 9a); this effect was less pronounced in the HLB-affected stem tissues (Fig. 9b), with less Zn distributed to phloem and epidermis tissues as compared to the control samples.

## Discussion

The use of enhanced nutritional programmes (ENPs) to minimize the deleterious effects of the HLB bacterial disease has been a topic of considerable discussion and debate since the discovery of HLB in Florida. While Gottwald *et al.* (2012) suggested that the ENPs did not sustain tree health, yield, or fruit quality of HLB-symptomatic trees, a survey of mandarin trees grown in different soil types and given different fertilizer regimes showed that applications of a foliar fertilizer (containing N fertilizer and minerals) reduced symptom expression of HLB-infected trees by about 40%, whereas fertilizers provided through soils did not have the same ameliorative effect (Pustika *et al.*, 2008). This suggested that Las infection restricted nutrient uptake and/or transport and, therefore, that foliar-applied minerals might prolong tree life and reduce yield losses (Pustika *et al.*, 2008). It has been reported that the concentrations of Fe and Zn in HLB-affected citrus plants were, on average, approximately half of those in healthy plants and Mn was occasionally reduced in infected plants (Koren *et al.*, 2013). The application of Zn or Cu ions in combination with Ca was able to delay HLB incidence and severity (Webb, 2006). However, there have also been reports that there was no relationship between nutritional deficiency status and HLB incidence in citrus and that Zn was significantly higher in HLB-affected trees, even though the mineral nutrient treatments provided some stress relief for Las-infected plants (Razi *et al.*, 2011). The results from the present study clearly show that Las infection results in significantly reduced levels of Zn and several other mineral nutrients in the tissues of grapefruit trees (Table 1).

Comparative µ-XRF imaging analyses of mineral element distributions in healthy and HLB-affected plant tissues suggest that a reduced phloem transport of Zn is one of the important factors that contribute to HLB-induced Zn deficiency in grapefruit. Firstly, the preferential localizations of Zn to phloem tissues in healthy leaves (Fig. 5a) and stems (Fig. 8a) in the present study suggest that, in grapefruit plants, this micronutrient is largely remobilized by phloem transport. To the best of our knowledge, this is the first indirect evidence for high Zn mobility in the phloem of citrus. Zinc was considered to be a mineral element with intermediate phloem mobility (Marschner, 1995). A previous report (Storey and Treeby, 2000) also demonstrated a moderate or low mobilization of Zn in citrus; for example, in navel orange trees, Zn mobility by phloem was very low during fruit development. Since minerals with high mobility in growth sinks may come from phloem remobilization (Marschner, 1995), the preferential localization of Zn to phloem tissues implies that phloem remobilization of Zn may contribute considerably to Zn density in the young leaves of healthy grapefruit plants. The absence of preferential Zn localization to the phloem tissues of HLB-affected leaves (Fig. 5b) and stems (Fig. 8b), compared with healthy plants, strongly suggested that phloem remobilization of Zn in grapefruit trees was largely limited by Las infection, and thus results in significantly decreased Zn concentrations in the young and mature leaves from HLB-affected grapefruit (Table 1).

The reduced phloem remobilization of Zn to growth sinks (young leaves) is presumably the result of HLB-induced disruption of phloem systems in stems and leaf petioles. The main cause of HLB symptoms in citrus is the disruption of phloem, which blocks the source–sink flow of photosynthates and nutrients. The HLB bacterium resides within the phloem’s sieve tube elements and is transmitted by Asian citrus psyllids that feed on the phloem sap of infected plants (Gottwald, 2010). It has been reported that one of the first degenerative changes induced upon invasion of the Las bacterium is the swelling of the middle lamella between the cell walls of phloem sieve elements (Folimonova and Achor, 2010). The subsequent HLB-associated phloem blockage apparently results from a plant-based response to infection (i.e. callose deposition) that causes the plugging of sieve plate pores rather than obstruction by aggregates of ‘*Ca.* L. asiaticus’ cells, since the HLB-causing pathogen does not form aggregates in citrus (Kim *et al.*, 2009). Koh *et al.* (2012) has suggested that callose deposition disrupts phloem transport of carbohydrates and minerals such as Zn, thus contributing to the development of HLB symptoms. The present results provide strong and specifically localized support for this correlation between HLB-infection, disruption of phloem, and reduced Zn remobilization to growth sinks (young leaves).

Zinc deficiency symptoms are very similar to those of HLB (Cevallos-Cevallos *et al.*, 2011), such as leaf chlorosis and stunted plant growth, which was also observed for HLB-affected grapefruit by the present study. The appearance of chlorotic symptoms in leaves of HLB-affected trees has been correlated to the disruption of phloem translocation of carbohydrates during infection (Folimonova *et al.*, 2009), presumably as a result of the chloroplast disintegration caused by excessive starch accumulation (Etxeberria *et al.*, 2009). The results from the present study demonstrate the coincidence of HLB symptoms, phloem disruption, and reduced Zn accumulation and transport when infected grapefruit trees are examined 12 months after graft-inoculation with Las. Earlier post-inoculation examination of infected trees might indicate whether the reduced Zn transport implied by our ICP-MS and µ-XRF analyses follows or precedes the well-known HLB-related phloem disruption. It is easy to suggest that phloem disruption starves roots of carbohydrate for use in energy and carbon-compound synthesis and that, in turn, causes the reduced uptake of Zn and other mineral elements from the soil. However, the above-average levels of starch accumulation in citrus leaves and branches observed under Zn deficiency (Etxeberria *et al.*, 2009; Smith, 1974) suggests a linkage of Zn with the phloem system’s function in the absence of HLB. Thus, the mechanisms involved must be investigated further in order to understand HLB disease-induced mineral deficiencies better, and Zn deficiency in particular, in order to develop an optimized strategy for managing HLB-affected citrus, a strategy that could include ENPs.

## Supplementary data

Supplementary data can be found at *JXB* online.

Supplementary Fig. S1. Micro-XRF mapping of elements (Fe, Mn, Cl, S, and P) in the cross-sections of young, mature and old leaves collected from healthy (a) and HLB-affected (b) grapefruit plants. Scale bar: 400 µm.

Supplementary Data

## Abbreviations:

HLB: Huanglongbing
µ-XRF: micro-X-ray radiation fluorescence.
