# Supplementary material for: Spatial imaging of Zn and other elements in Huanglongbing-affected grapefruit by synchrotron-based micro X-ray fluorescence investigation
Source: J Exp Bot. 2014 Jan 13;65(4):953–64. doi: 10.1093/jxb/ert450 (PMC3935563; doi:10.1093/jxb/ert450)
Supplement: Supplementary Data [file supp_65_4_953__index.html]

Spatial imaging of Zn and other elements in Huanglongbing-affected grapefruit by synchrotron-based micro X-ray fluorescence investigation — Spatial imaging of Zn and other elements in Huanglongbing-affected grapefruit by synchrotron-based micro X-ray fluorescence investigation — Supplementary Data 

# Spatial imaging of Zn and other elements in Huanglongbing-affected grapefruit by synchrotron-based micro X-ray fluorescence investigation

## Supplementary Data

Data files

**Files in this Data Supplement:**

- Supplementary Data - Supplementary Data
