# Supplementary material for: Spatial imaging of Zn and other elements in Huanglongbing-affected grapefruit by synchrotron-based micro X-ray fluorescence investigation
Source: J Exp Bot. 2014 Jan 13;65(4):953–64. doi: 10.1093/jxb/ert450 (PMC3935563; doi:10.1093/jxb/ert450)

Journal of Experimental Botany

Spatial imaging of Zn and other elements in Huanglongbing-affected grapefruit by microscopically focused synchrotron X-ray investigation. *Shengke Tian, Lingli Lu, John M. Labavitch, Samuel M. Webb, Xiaoe Yang, Patrick H. Brown, and Zhenli He.*

Supplementary Data

Figure S1. Micro-XRF mapping of elements (Fe, Mn, Cl, S, and P) in the cross-sections of young, mature and old leaves collected from healthy (a) and HLB-affected (b) grapefruit plants. Scale bar: 400 µm.

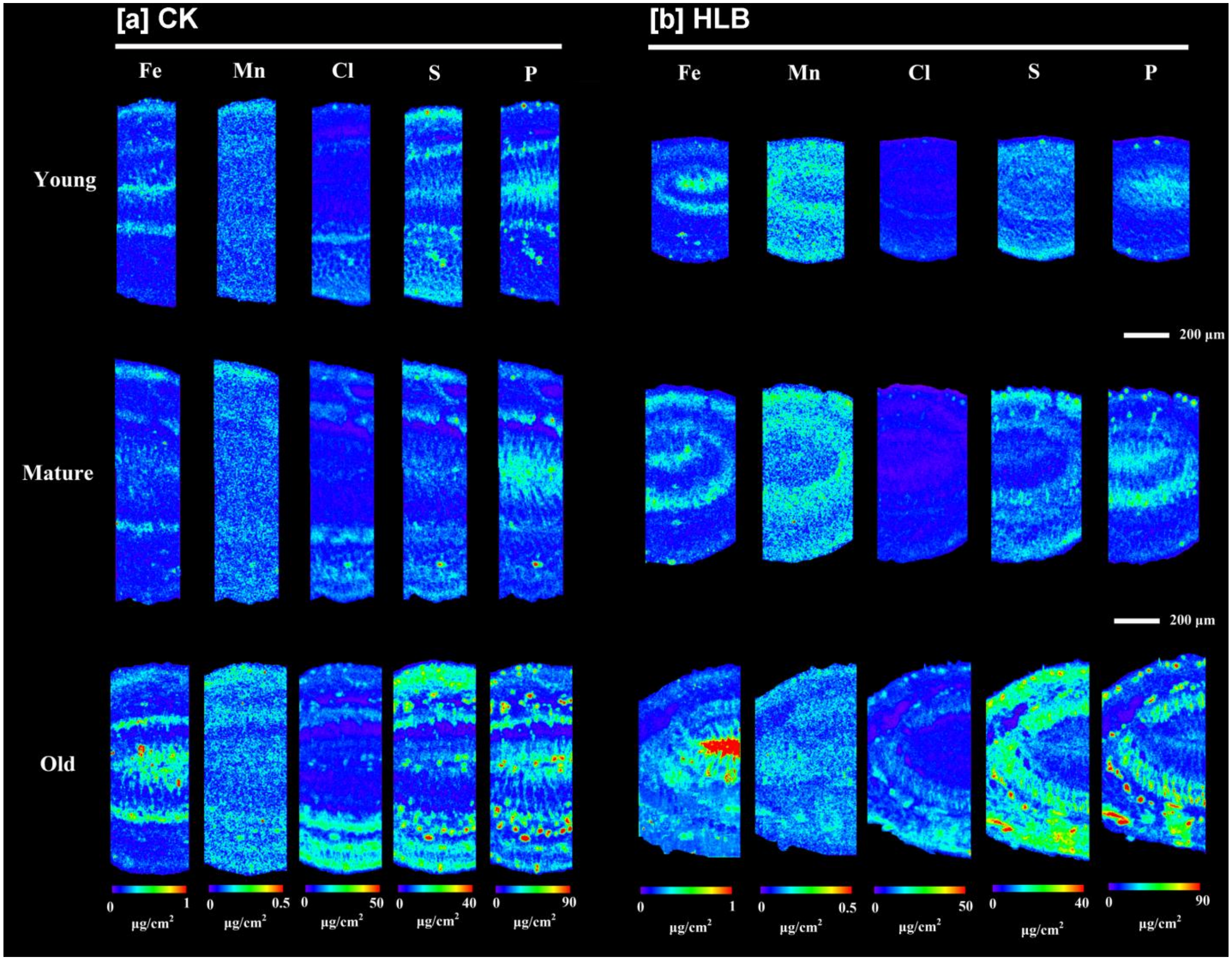

Supplement: Supplementary Data [file supp_ert450_jexbot107581_file001.pdf]
